# Osteoclasts Are Active in Bone Forming Metastases of Prostate Cancer Patients

**DOI:** 10.1371/journal.pone.0003627

**Published:** 2008-11-03

**Authors:** Ilaria Roato, Patrizia D’Amelio, Eva Gorassini, Anastasia Grimaldi, Lisa Bonello, Cristian Fiori, Luisa Delsedime, Alessandro Tizzani, Alfredo De Libero, Giancarlo Isaia, Riccardo Ferracini

**Affiliations:** 1 CeRMS (Center for Experimental Research and Medical Studies) University and A.O.U. San Giovanni Battista, Turin, Italy; 2 Department of Medical Oncology2, University and A.O.U. San Giovanni Battista, Turin, Italy; 3 Department of Internal Medicine, University and A.O.U. San Giovanni Battista, Turin, Italy; 4 Department of Urology, University and A.O.U. San Giovanni Battista, Turin, Italy; 5 Department of Pathology, A.O.U. San Giovanni Battista, Turin, Italy; 6 Department of Orthopaedics, A.O.U. San Giovanni Battista, Turin, Italy; Ordway Research Institute, United States of America

## Abstract

**Background:**

Bone forming metastases are a common and disabling consequence of prostate cancer (CaP). The potential role of osteoclast activity in CaP bone metastases is not completely explained. In this study, we investigated *ex vivo* whether the osteolytic activity is present and how it is ruled in CaP patients with bone forming metastases.

**Methodology:**

Forty-six patients affected by newly diagnosed CaP and healthy controls were enrolled. At diagnosis, 37 patients had a primary tumour only, while 9 had primary tumour and concomitant bone forming metastases. In all patients there was no evidence of metastasis to other non-bone sites. For all patients and controls we collected blood and urinary samples. We evaluated patients' bone homeostasis; we made peripheral blood mononuclear cell (PBMC) cultures to detect *in vitro* osteoclastogenesis; we dosed serum expression of molecules involved in cancer induced osteoclatogenesis, such as RANKL, OPG, TNF-alpha, DKK-1 and IL-7. By Real-Time PCR, we quantified DKK-1 and IL-7 gene expression on micro-dissected tumour and healthy tissue sections.

**Principal Findings:**

CaP bone metastatic patients showed bone metabolism disruption with increased bone resorption and formation compared to non-bone metastatic patients and healthy controls. The CaP PBMC cultures showed an enhanced osteoclastogenesis in bone metastatic patients, due to an increase of RANKL/OPG ratio. We detected increased DKK-1 serum levels and tissue gene expression in patients compared to controls. IL-7 resulted high in patients' sera, but its tissue gene expression was comparable in patients and controls.

**Conclusions:**

We demonstrated *ex vivo* that osteoclastogenesis is an active mechanism in tumour nesting of bone forming metastatic cancer and that serum DKK-1 levels are increased in CaP patients, suggesting to deeply investigate its role as tumour marker.

## Introduction

Prostate cancer (CaP) is the second leading cause of cancer-related deaths in men [1]. A typical feature of CaP is the ability to metastatize to the bone, as more than 80% of men who died for CaP developed bone metastases [2]. Growth of CaP within bone promotes primarily osteoblastic lesions with underlying osteolytic areas [1]. In physiological conditions osteoclast (OC) and osteoblast activity is well balanced, but in pathologic condition this equilibrium (bone homeostasis) is disrupted. The role of OCs in osteolytic metastases has been clearly demonstrated, and it has been suggested that OCs might play an important role in the osteoblastic metastases as well. Interactions among CaP cells, osteoblasts and OCs suggest that bone tropism might be caused by tumour dependence on one or more osteoblast/OC–derived factors for cancer cell growth and maintenance [3].

We previously demonstrated that patients with osteolytic bone metastases from different solid tumours showed enhanced spontaneous osteoclastogenesis, due to increased T cell release of pro-osteoclastogenic factors, such as TNF-alpha, IL-7 and RANKL [4]. The role of OPG/RANKL axis in the osteolytic component of CaP-mediated bone metastases has been elucidated [5], while TNF-alpha and IL-7 possible functions in CaP bone lesions remain unclear.

Other key factors in bone biology are WNTs, a family of small secreted glycoproteins, which inhibits OC differentiation [6], stimulates osteoblastogenesis and mineralizing activity of osteoblasts [7]. The activity of WNT family is antagonized by several secreted factors such as dickkopf (DKK) proteins. Recent data reported DKK-1 expression in some human specimens of tumours, suggesting that a cancer-mediated modulation of WNT activity influences the metastatic phenotype [8], [9].

This cross-sectional investigation was designed to study how bone forming metastases by CaP affects bone turnover, OC formation by peripheral blood mononuclear cells (PBMC), and the production of osteoclastogenic and anti-osteoclastogenic factors in patients affected by bone metastatic CaP.

We report an increased osteoclastogenesis in CaP bone metastatic patients, due to an increase in the serum RANKL/OPG ratio, suggesting that enhanced OC formation plays an active role in bone forming metastases. We detected high DKK-1 serum levels and gene expression in CaP patients compared to healthy controls.

## Results

### Bone turnover is increased in bone metastatic patients

The markers of bone turnover were higher in patients with bone metastases compared to non-bone metastatic patients and healthy controls (Table 1). In detail, CaP patients did not show significant differences in bone density, but had higher PTH, BAP, BGP, TRAPC5b and crosslink levels than healthy controls. These results confirm the disruption in bone homeostasis with increased bone resorption and formation in metastatic patients.

**Table 1:** Characteristics of patients and healthy controls.

	Patients without bone metastases (37)	Patients with bone metastases (9)	Healthy controls (20)	*p*
**Age ** ***(yrs)***	64±7	67±10	60±6	NS
**BMI**	25.9±2.4	25.9±2.4	25.4±2.3	NS
**Lumbar BMD ** ***(g/cm^2^)***	1.02±0.1	1.06±0.1	1.03±0.2	NS
**Femoral neck BMD ** ***(g/cm^2^)***	0.73±0.1	0.75±0.1	0.76±0.1	NS
**PTH ** ***(pg/ml)***	50.15±22.6	69.8±34.3*	34.4±15.9	0.018
**Calcium ** ***(mEq/L)***	4.6±0.2	4.2±1	4.6±0.2	NS
**Phosphate ** ***(mMol/L)***	1.02±0.1	1.13±0.1	1.07±0.2	NS
**BAP ** ***(UI/L)***	11.61±6.4	55.5±21.8*	10.7±4.4	0.001
**BGP ** ***(ng/ml)***	4.6±2.4	19.6±8.9*	5.00±2.2	0.000
**TRAP5b ** ***(U/L)***	2.1±0.4	7.8±6.5*	2.4±0.6	0.001
**Cross links ** ***(nM/mM creat)***	5.49±1.5	15.4±4.1*	6.7±3.3	0.001

Bone turnover marker values are shown as mean±SD, the p values were calculated by one way ANOVA and the Bonferroni post-hoc correction. * and ° indicates the values significantly different between patients with/ without bone metastases (* *p* = 0.001, ° *p* = 0.000).

### Osteoclastogenesis is increased in CaP bone metastases

To evaluate whether the enhancement of bone resorption in metastatic patients is due to an increase in OC formation, we examined the ability of *in vitro* PBMCs to spontaneously differentiate in OCs in patients with or without bone metastases and in healthy controls.

**Figure 1:**
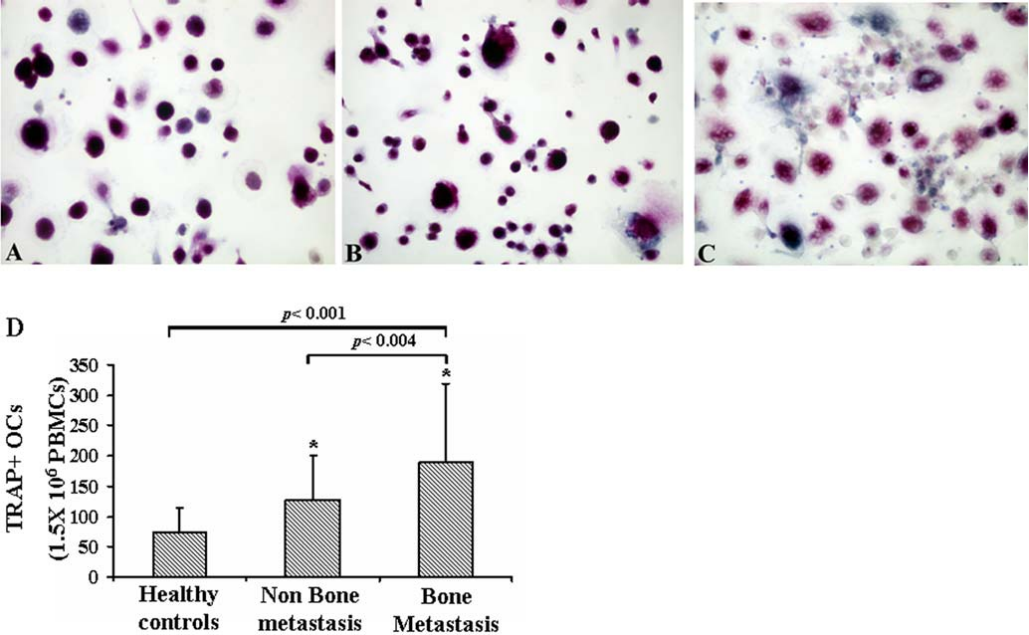
Analysis of osteoclastogenesis from CaP patients' PBMCs. TRAP positive multinucleated cells were identified as OCs and counted, in both patients and healthy controls cultures, (A). The OC number in bone metastatic patients was significantly higher than in non-bone metastatic patients, *p*<0.004 and in healthy controls, *p*<0.001 (B).

The OC differentiation was demonstrated by the presence of multinucleated/TRAP positive cells from cancer patient and healthy control PBMCs (Fig. 1A–C). As showed in Fig. 1D the number of OCs was significantly higher in bone metastatic patients (mean±se, 216.22±39.55) than in patients without bone metastases (112.71±14.76) and in healthy controls (73.55±11.69), *p*<0.001.

### RANKL/OPG ratio is increased in metastatic patients

In order to investigate the factors responsible for the increased osteoclastogenesis in patients, we dosed the serum levels of TNF-alpha, RANKL and OPG. The TNF-alpha serum levels were not significantly different among the three groups (data not shown), while we observed a significantly increased ratio RANKL/OPG in bone metastatic sera (19.62±6.52) compared to non-metastatic patients (5.48±2.48) and healthy controls (6.89±2.6), *p*<0.03.

### IL-7 serum level is increased in cancer patients

**Figure 2:**
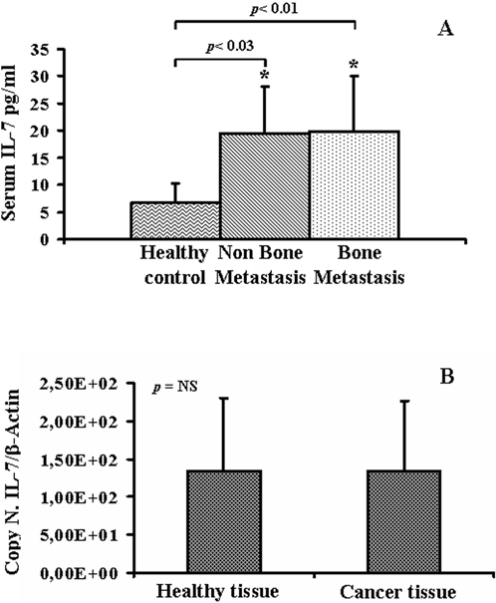
IL-7 expression by CaP. IL-7 serum levels in patients with/without bone metastases and in healthy controls were measured by ELISA. Bone metastatic (*p*<0.01) and non-bone metastatic patients (*p*<0.03) had significantly higher IL-7 serum levels compared to healthy controls (A). CaP and healthy tissues were analyzed by Real-Time PCR in order to quantify IL-7 gene expression. The IL-7 quantization was expressed as IL-7 on β-Actin (the control gene) plasmid copy number. The histogram showed comparable IL-7 expression levels in CaP and healthy tissues.

We measured IL-7 serum levels in patients and controls. Serum IL-7 levels were significantly higher in bone metastatic patients (mean±se, 19.86±2.01 pg/ml) than in healthy controls (7.07±1.27 pg/ml), p<0.001. We dosed comparable IL-7 levels in non-bone metastatic (19.75±3.55 pg/ml) and bone metastatic patients (19.86±2.01 pg/ml), (Fig. 2A). This result led us to investigate whether tumor cells were responsible for the increase of IL-7 production; therefore we examined the quantitative IL-7 expression in CaP and in healthy prostate tissues. Tumour cells expressed low and comparable levels of IL-7 in patients and healthy controls (Fig. 2B). This suggests that the increased circulating IL-7 might depend on the production by the immune system cell, such as T and B lymphocytes [4].

### DKK-1 expression is higher in CaP patients

**Figure 3:**
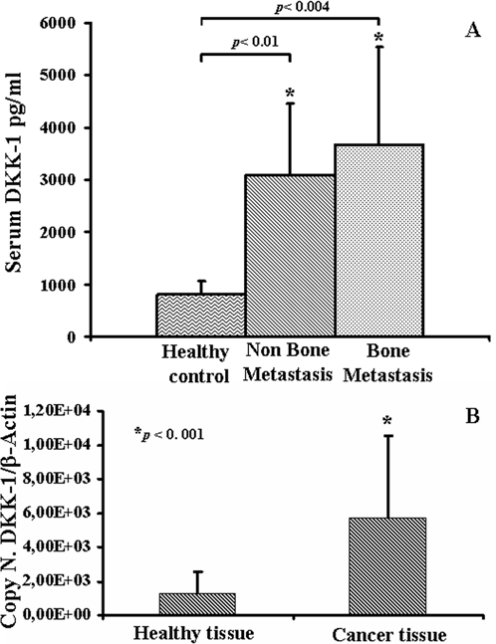
DKK-1 expression is higher in CaP patients. DKK-1 levels were dosed in serum patients with/without bone metastases and in healthy controls by ELISA. Bone metastatic (*p*<0.004) and non-bone metastatic patients (*p*<0.01) had significantly higher DKK-1 serum levels compared to healthy controls (A). CaP and healthy tissues were analyzed by Real-Time PCR in order to quantify DKK-1gene expression. The DKK-1 quantization was expressed as DKK-1 on β-Actin (the control gene) plasmid copy number. The histogram showed higher DKK-1 expression levels in CaP than in healthy tissues, *p*<0.001 (B).

Literature data reported that DKK-1 is involved in bone homeostasis [8]. We dosed DKK-1 serum level in CaP patients and healthy controls. CaP patients showed higher DKK-1 levels than healthy controls, *p*<0.004 (Fig. 3A). To evaluate whether or not DKK-1 is produced by cancer tissues, we studied its expression on CaP and healthy tissues by RQ-PCR. Our data demonstrated that CaP tissue expressed significantly more DKK-1 than healthy tissue, p<0.001 (Fig. 3B).

## Discussion

Prostate cancer frequently develop bone forming metastases; nonetheless a brisk osteolytic activity is present in metastatic compared to non-metastatic patients [10]. The mechanisms through which CaP promotes aberrant bone remodelling are not clearly defined. The disclosure of tumour nesting in bone might provide new tools for an early diagnosis of bone metastases and suggest novel therapeutic regimens for the control of CaP progression. The aim of our study was to investigate how the osteolytic component of bone metastasis affects bone turnover, OC formation by PBMC, and the production of osteoclastogenic and anti-osteoclastogenic factors in patients affected by bone metastatic CaP.

In the selection of CaP cases, we decided to avoid patients with an advanced metastatic disease, since therapeutic regimens might represent a bias for our analysis. In this study, we have an imbalance between the bone and non-bone metastatic patients' number, which depends on the presence of a smaller amount of bone metastatic compared to non-bone metastatic CaP patients at diagnosis [3]. In fact, in the natural history of the disease, bone metastases are a frequent, but late event [11].

The bone turnover was enhanced in bone metastatic patients, in particular we observed both an increase in bone formation and resorption markers. PTH level was slightly increased in bone metastatic patients compared to healthy controls, according to PTH ability to promote the growth and invasiveness of prostate cancer cells in bone [12].

The observed increase in bone resorption and the previously demonstrated spontaneous osteoclastogenesis in cancer patients with osteolytic metastases [13] prompted us to investigate osteoclastogenesis from CaP patients' PBMC *in vitro*. OC formation was higher in bone metastatic patients compared to both non-bone metastatic patients and healthy controls. To identify the factors responsible for the increase in OC formation, we measured molecules mostly involved in osteoclastogenesis, such as TNF-alpha, RANKL, OPG, IL-7 and DKK-1. The TNF-alpha serum levels were not significantly increased in CaP patients, differently from other bone metastatic tumours, where TNF-alpha plays an important role in osteoclastogenesis [14]. Otherwise the RANKL/OPG ratio was higher in bone metastatic patients, explaining the increased osteoclastogenesis and according to previous literature data [15].

The interplay among the tumour cells, the immune system and the bone tissue has become a relevant object of intensive study. Since IL-7 involvement in bone metastasis was previously demonstrated in other tumours [4], [16], we investigated this issue showing an increase in serum IL-7 levels in CaP patients with and without bone lesions. The increase of IL-7 might account for the RANKL/OPG augment, since IL-7 stimulates RANKL production from T cells [17]. We evaluated IL-7 gene expression in CaP and normal prostate tissues, showing comparable IL-7 expression in prostate cancer and normal tissues. This result differs from our published data on lung cancer, where the tumour tissue expressed higher IL-7 levels compared with the normal counterpart [18]. We suggest that this discrepancy might be due to the different tumour type and bone metastatic behaviour, as lung cancer causes osteolytic metastases, while CaP produces mainly bone forming lesions. The increased IL-7 serum level may depend on immune system activation against the tumour. In fact, it has been previously demonstrated that T and B cells produce IL-7, in both tumours and other pathologies associated to bone resorption [4], [19], [20]. WNT signalling plays an important role in bone development, since it inhibits OC differentiation [6], stimulates osteoblastogenesis and mineralizing activity of osteoblasts [7]. WNT proteins are also expressed by CaP and can promote tumour bone invasion [21]. DKK is a soluble inhibitor of canonical WNT signalling [22]. A recent study associates DKK-1 expression in breast cancer with the presence of bone metastases [23]. Data regarding DKK-1 expression in CaP are scant: some authors report an increase DKK-1 expression in osteolytic lesions, but not in the primary tumours [8]. Hall *et al* reported that CaP-derived DKK-1 is involved in osteoblastic activity in bone metastases, since DKK-1 signalling might account for switching the bone response to CaP cells from osteolytic to osteoblastic and vice versa [24]. In this work, we studied only patients with bone forming metastases, therefore we are unable to correlate osteolytic activity induced by CaP cells and DKK-1 expression, as previously described [8]. Nonetheless, we found a large amount of DKK-1 in serologic samples from cancer patients and an enhanced DKK-1 gene expression in CaP tissues, suggesting that the increased serum DKK-1 levels in CaP patients might depend on the CaP cell secretion. This result will be deeply study in order to evaluate the potential role of DKK-1 as tumour marker in CaP. Moreover, we could speculate that CaP cells stimulate bone marrow environment to increase the DKK-1 release through unknown mechanisms. In our bone metastatic patients, serum DKK-1 levels are slightly increased compared to non-metastatic patients, without a statistically significant difference. This could depend on our low number of patients, but investigating a large number of patients, we expect to show a difference between the two groups, confirming the literature data [23].

## Materials and Methods

### Patients and markers of bone turnover

The experimental project and all the studies performed on the patients were approved by the Ethical Committee of our Institution (Azienda Ospedaliera–Universitaria San Giovanni Battista in Torino) and written informed consent from patients and healthy controls was obtained. The studied population included 46 patients affected by newly diagnosed CaP (37 had a primary tumour only, while 9 had primary tumour and concomitant bone forming metastases) and 20 healthy men. In all patients there was no evidence of metastasis to other non-bone sites. It has been demonstrated that estrogen loss significantly affect osteoclast formation [25]. Thus we studied CaP that, being an only male tumour, avoids by default all the possible biases due to the cyclical estrogen variations and postmenopausal fall in estrogen levels in females. Patients and controls were matched for age and body mass index. Bone mineral density (BMD) was measured by double-emission X-ray absorptiometry with a Hologic QDR 4500 at lumbar spine and femoral neck both in patients and controls. Subjects with intestinal malabsorption diseases, other kind of deficient nutritional status, secondary osteoporosis or taking drugs active on bone turnover or anti cancer therapy were excluded. The presence of bone metastases was confirmed using ^99^Tc bone scanning and further imaging studies according to the standard clinical practice.

In order to investigate bone metabolism status, patients and controls were subjected to analysis of standard clinical markers of bone metabolism, such as serum PTH, bone alkaline phosphatase (BAP), calcium, phosphate, osteocalcin (BGP) and urinary deoxypyridinoline (urinary crosslinks) [26]. In particular, crosslinks dosage has been chosen in clinical practice to monitor bone metastatic disease and the response to anti-resorbing treatments such as bisphosphonates [27], [28]. As markers of bone resorption we also measured Tartrate Resistent Acid Phosphatase 5b (TRAP5b) in sera [29], by BoneTRAP ELISA kit (Suomen Bioanalytiikka Oy, Turku, Fin). Serological markers, such as PSA and histological grading, according to Gleason, were recorded for all the patients included in this study [30], [31]. All biochemical measurements were performed on a single blood or urinary sample at a single time point per subject.

### Cell cultures

As previously described [13], for all patients and healthy controls, PBMCs were isolated from peripheral blood and cultured in α-MEM, supplemented with 10% FBS, penicillin 100 U/ml and streptomycin 100 µg/m (Cambrex, Bio Science, Walkersville, MD), without adding exogenous stimulatory factors such as M-CSF and RANKL. After 15 days, cultures were stopped, mature OCs were identified as multinucleated cells containing three or more nuclei and positive for TRAP expression (Sigma Aldrich, St. Louis, MO).

### Cytokines dosage

In order to evaluate factors involved in osteoclastogenesis the amount of serum total RANKL (free and OPG-bound), OPG, TNF-alpha, IL-7 and DKK-1 were determined by commercially available ELISA kit according to manufacturer's instructions. Samples were assayed in duplicate and data were expressed as mean values. The sensitivities were: 1.56 to 30000 pg/ml for total RANKL (Apotech Corporation, Epalinges, CH); 0 to 4000 pg/ml for OPG; 0.12 to 32 pg/ml for TNF-alpha; 0.1 to 16 pg/ml for IL-7 (R&D system, Abingdon, UK) and 0.38 to 50 pmol/L for DKK-1 (Biomedica, Wien, A).

### RNA extraction from formalin-fixed, paraffin-embedded (FFPE) CaP tissues

We extracted RNA from FFPE CaP and healthy tissues. For 19 patients, we obtained samples from tumour and healthy tissue blocks, the tumour areas were selected on the haematoxylin/eosin sections by microscope and manually dissected. RNA was isolated from FFPE CaP tissue block (10 µm sections) using the RecoverAll Total Nucleic Acid Isolation Kit (Ambion, Huntingdon, UK). 1 µg of RNA was converted up to single stranded cDNA by the High-Capacity cDNA Reverse Transcription Kit (Applied Biosystems, Warrington, UK).

### Generation of patient plasmid standard

PCR primers for IL-7, DKK-1 and β-Actin were designed using Primer Express v2.0 software and synthesized by Applied Biosystems (Warrington, UK). The IL-7 and β-Actin sequences were previously published [18], while the DKK-1 sequences were: sense 5′-GGAAGCGCCGAAAACG-3′ and antisense 5′-ACACACATATTCCATTTTTGCAGTA-3′. Purified fresh PCR products of 80-bp for IL-7, 75-bp for DKK-1 and 78-bp for β-Actin were directly cloned using TA Cloning Kit Dual Promoter (Invitrogen, Carlsbad, CA) as previously described [18]. Positive clones were sequenced to confirm their identity. 10 µg of the selected plasmid for the genes were digested with 8 U of Hind III restriction enzyme overnight at 37°C. Linearized plasmids were finally purified with NucleoSpin clean up extraction kit (Macherey-Nagel, Düren, D), resuspended with 1× TE and OD_260_ was determined. Copy number was calculated from the plasmid concentration, mean molecular weight of the nucleotides (660 g/Mol) and plasmid plus insert length.

### Real-Time Quantitative analysis of IL-7 and DKK-1 gene expression

Considering the higher amount of serum IL-7 and DKK-1 in CaP patients both with and without bone metastases, we decided to investigate whether these factors are produced by tumor cells. We performed quantitative analysis of IL-7 and DKK-1 expression by Real-Time Quantitative PCR (RQ-PCR), β-Actin was the housekeeping control. RQ-PCR analysis of IL-7 and DKK-1 was carried out using the iCycler iQ™ system (Bio Rad, Hercules, CA, USA). TaqMan probes were designed using Primer Express v2.0 software and synthesized by Applied Biosystems (Warrington, UK). IL-7 and β-Actin specific TaqMan probes were previously used [18], while the DKK-1 probe was (5′-ATGCGTCACGCTATGTGCTGCC-3′). All the probes were labelled at the 5′ end with 6-carboxy fluorescein (FAM) and the 3′ end with 6-carboxy-tetrametil rhodamine (TAMRA). Reactions for IL-7, DKK-1 and β-Actin quantification were performed in a 25 µl final volume with 2 µl of sample cDNA, 1× iQ Supermix (Bio Rad, Hercules, CA, USA), 0,3 µM of each primer and 0,4 µM of the probes. PCR primers were the same used for IL-7, DKK-1 and β-Actin cloning. The amplification conditions for quantization were: 95°C for 15 minutes, 50 cycles at 95°C for 15 seconds, 58°C for IL-7, 60°C for DKK-1 and β-Actin for 1 minute.

### Statistical analyses

Statistical analyses were performed by the Statistical Package for the Social Sciences (spssx/pc) software 15.0 (SPSS, Chicago, IL, USA). Continuous variables were expressed as mean and standard deviation. In order to compare patients both with and without bone metastases and controls, we performed a one way analysis of variance (ANOVA). Bonferroni correction was carried out for multiple comparisons. To explore the effect of the continuous variables analysed on OC formation, we fitted a linear regression model by step way procedure. The gene expression of DKK-1 and IL-7 was compared between tumour and healthy tissue by paired Student's T test. The results were considered statistically significant for *p*<0.05.
